# Motorboat noise disrupts co-operative interspecific interactions

**DOI:** 10.1038/s41598-017-06515-2

**Published:** 2017-08-01

**Authors:** Sophie L. Nedelec, Suzanne C. Mills, Andrew N. Radford, Ricardo Beldade, Stephen D. Simpson, Brendan Nedelec, Isabelle M. Côté

**Affiliations:** 1 School of Biological Sciences, Life Sciences Building, 24 Tyndall Avenue, University of Bristol, BS8 1TQ Bristol, UK; 2 EPHE PSL Research University, USR 3278 CRIOBE CNRS-UPVD, CRIOBE BP 1013 Móorea, 98729 Polynesie Francaise, France; 3 Laboratoire d’Excellence “CORAIL”, Perpignan, France; 4 Biosciences, College of Life and Environmental Sciences, University of Exeter, Geoffrey Pope, Stocker Road, Exeter, EX4 4QD UK; 5 Earth to Ocean Group, Department of Biological Sciences, Simon Fraser University, Burnaby, BC V5A 1S6 Canada

## Abstract

Human-made noise is contributing increasingly to ocean soundscapes. Its physical, physiological and behavioural effects on marine organisms are potentially widespread, but our understanding remains largely limited to intraspecific impacts. Here, we examine how motorboats affect an interspecific cleaning mutualism critical for coral reef fish health, abundance and diversity. We conducted *in situ* observations of cleaning interactions between bluestreak cleaner wrasses (*Labroides dimidiatus*) and their fish clients before, during and after repeated, standardised approaches with motorboats. Cleaners inspected clients for longer and were significantly less cooperative during exposure to boat noise, and while motorboat disturbance appeared to have little effect on client behaviour, as evidenced by consistency of visit rates, clientele composition, and use of cleaning incitation signals, clients did not retaliate as expected (i.e., by chasing) in response to increased cheating by cleaners. Our results are consistent with the idea of cognitive impairments due to distraction by both parties. Alternatively, cleaners might be taking advantage of distracted clients to reduce their service quality. To more fully understand the importance of these findings for conservation and management, further studies should elucidate whether the efficacy of ectoparasite removal by cleaners is affected and explore the potential for habituation to boat noise in busy areas.

## Introduction

Anthropogenic (human-made) noise is contributing increasingly to soundscapes on a global scale. The extent to which excessive noise has an impact on animals is of increasing concern and, in some regions, noise disturbance is now regulated by local, national and international legislation (e.g., US National Environment Policy Act and European Commission Marine Strategy Framework Directive)^1^. Sounds produced by human activities can cause a range of physical, physiological and behavioural effects in a wide range of taxa, including mammals, birds, reptiles, amphibians, fishes and invertebrates^2, 3^. Behavioural impacts on fish include increased hiding^4, 5^, lower frequencies of feeding and egg-caring behaviour^6, 7^, increased movement and displacement^8^, impaired orientation^9^, and poorer anti-predator responses^10, 11^. To our knowledge, all studies but one to date has focused on the responses of individual species. The single exception showed that prey fish responded more slowly to simulated attacks under noisy than quiet conditions, leading to enhanced hunting success by predators^11^. As with predator-prey relationships, cooperative mutualisms can also influence population dynamics^12^, but the impacts of anthropogenic noise on these types of interactions have not yet been considered.

Cleaning relationships are one of the most iconic of cooperative interspecific interactions among marine fishes. They typically entail a small fish – the cleaner – removing ectoparasites and other items from the body surface, buccal cavity or gills of a larger fish – the client. Cleaning interactions are ubiquitous and a large number of species take part, particularly on coral reefs^13^. The interactions involving some cleaners, such as cleaning gobies (*Elacatinus* spp.), are almost invariably cooperative since those cleaners prefer to forage on ectoparasites^14^. However, in cleaner wrasses (*Labroides* spp.), which prefer client mucus over ectoparasites^15^, the temptation to cheat (i.e., take items other than ectoparasites) is great and honesty is effectively enforced through various control strategies by clients^16, 17^. Cleaners compete for access to fish clients and clients are choosy, preferring cleaners that offer high-quality (i.e., honest, cooperative) cleaning services^18, 19^. Client species with large home ranges and access to several cleaners withhold further visits to cleaners that do not provide a good service, and switch to more honest partners^20^. By contrast, resident clients with access to only one cleaner punish dishonest cleaners with aggressive chases^21^, which results in greater honesty by cleaners in subsequent encounters^16^. Cheating bites by cleaners are easily detected by observers (humans and fish alike)^22^, because they elicit jolts by clients, i.e. short twitches of the client’s body in response to mouth contact by a cleaner^21^. Cleaner wrasses can significantly reduce the parasite loads of their clients^23^, and are deemed to be keystone species on coral reefs because their absence results in marked reductions in fish abundance and diversity^24, 25^.

There are reasons to expect that cooperative interspecific interactions such as cleaning mutualisms could be affected by anthropogenic noise. First, the effects of noise can vary among species. For example, playbacks of ship noise enhanced anti-predator response in three-spined sticklebacks *Gasterosteus aculeatus* but not in European minnows *Phoxinus phoxinus* ^26^. The behaviour of the multiple parties involved in cleaning mutualisms could therefore shift differently, causing miscommunication during interactions. Second, noise is known to affect social behaviour, at least within species. Cooperatively breeding cichlids *Neolamprologus pulcher* engaged in less cooperative egg defence, and dominant individuals increased aggression towards subordinates when exposed to boat noise^27^. In schooling fishes, anthropogenic noise can increase^28^ or decrease group cohesion^29^. Finally, exposure to human-made noise can result in poor decision-making, such as food handling errors (three-spined sticklebacks)^30^, or decreased flight distance from potential predators (eels *Anguilla anguilla*)^10^. Taken together, these observations suggest that cooperative cleaning interactions, which rely heavily on cognition, memory and decision-making^31, 32^, could potentially be impaired by noise.

Our goal was to examine the impact of anthropogenic noise on the interactions between cleaners and their fish clients. More specifically, we examined how the cleaning interactions of juvenile bluestreak cleaner wrasse *Labroides dimidiatus* (which, like adults of the species, provide cleaning services from small, permanent home ranges known as cleaning stations)^33^ are affected by short-term exposure to the noise of small outboard motorboats at Moorea Island. Motorboats are the most common source of anthropogenic noise in shallow reef environments^34^. We measured sound pressure and particle motion of diving observers and passing motorboats in proximity to cleaning stations to establish levels of acoustic exposure. In a preliminary experiment we tested for any potential habituation effects to diver presence by cleaners and clients. The objectives of our main experiment were to compare, before, during and after exposure to motorboat noise: (1) the willingness of clients to seek the services of cleaner wrasses – as measured by consistency of clientele composition, visit rates and propensity to adopt incitation postures^35^; (2) the willingness of cleaner wrasses to engage in cleaning – as measured by inspection duration; and (3) overall cooperation – as measured by client jolts, which reflect cheating bites by cleaners^21^, and retaliatory chases by their clients.

## Results

### Relative noise of diver and motorboat

**Figure 1:**
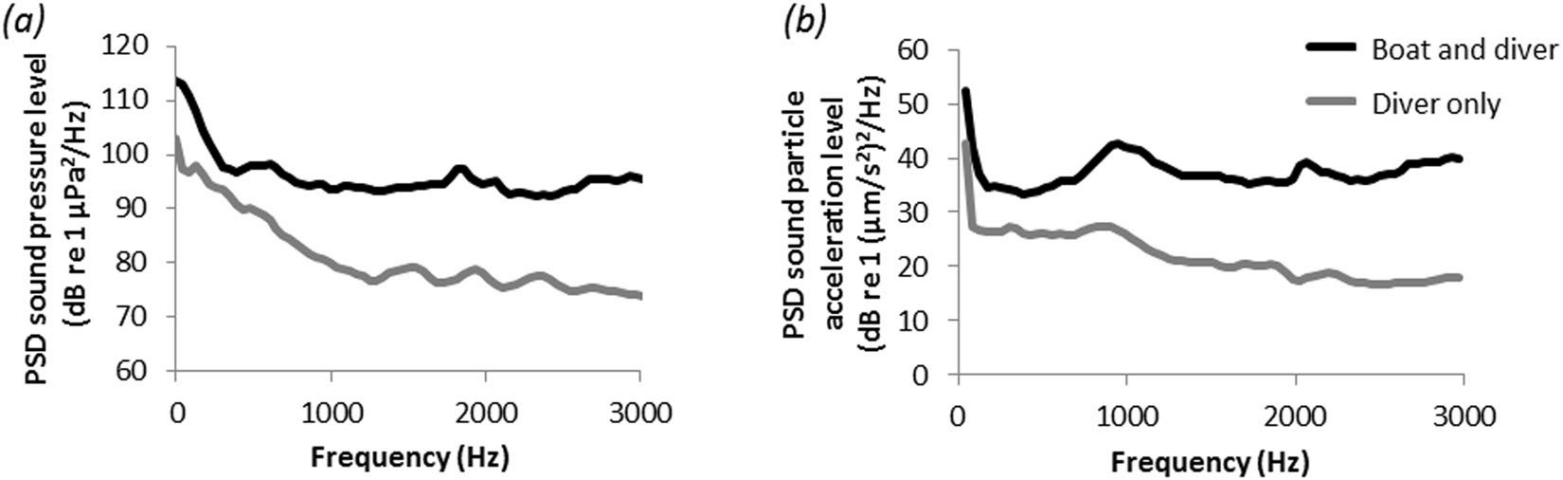
Mean power spectral density (PSD) levels for (**a**) sound pressure and (**b**) mono (horizontal) axis particle acceleration, over 5 min. Recordings were made within 50 cm of a juvenile bluestreak cleaner wrasse at 4 m depth on a coral wall with a SCUBA diver hovering motionless 1–2 m away. The grey line shows the noise caused by the diver; the black line, the noise caused by the diver and a motorboat passing repeatedly 10–100 m away from the cleaner and diver. Sample rate = 44.1 kHz, FFT length (number of frequency bands) = 1024.

Overall, the passage of a motorboat near the cleaning sites generated louder noise, both in terms of sound pressure (Fig. 1a) and particle acceleration (Fig. 1b), than a diver alone. The power spectral density (PSD) levels of a diver alone and those of the diver and motorboat combined both peaked at low frequencies (<100 Hz), but PSD levels for the combined motorboat and diver noise exceeded those of a diver alone across all frequencies (Fig. 1). For clarity, Fig. 1 shows only mean PSDs; spectrograms of sound recordings and plots of median, 5% and 95% exceedance levels (percentiles) are shown in Figures S1–S8.

### Habituation to diver noise and presence

We found no evidence of significant changes in cleaner or client behaviour over three consecutive 20-min periods in the presence of a diving observer in the absence of motorboat. The numbers of visiting clients were similar across three periods, as was total inspection duration (Wilcoxon signed-rank tests, all pairwise comparisons of periods, V < 34, n = 12, p > 0.13). There was also no significant difference in clientele composition among the 20-min periods (ANOSIM, R = −0.065, n = 12, p = 0.99; all pairwise comparisons, p > 0.72).

### Effects of motorboat noise on cleaner–client interactions

#### Willingness of clients to seek cleaning

There was no significant difference in composition of clientele at cleaning stations before, during and after exposure to motorboat noise (ANOSIM, R = −0.22, p = 0.99; all pairwise comparisons, p > 0.90), although there were significant differences among some sites (ANOSIM, R = 0.38, p = 0.004), reflecting spatial heterogeneity in reef fish assemblages. The total number of visiting clients per station did not vary significantly over time (Wilcoxon signed-rank tests, all pairwise comparisons of periods, V < 96.5, n = 21–24, p > 0.35). Moreover, the proportion of interactions initiated by clients, as indicated by client posing, was relatively constant, around 12%, across the three observation periods (V < 42, n = 21–24, p > 0.10 for all pairwise comparisons of periods).

#### Willingness of cleanerfish to engage in cleaning

**Figure 2:**
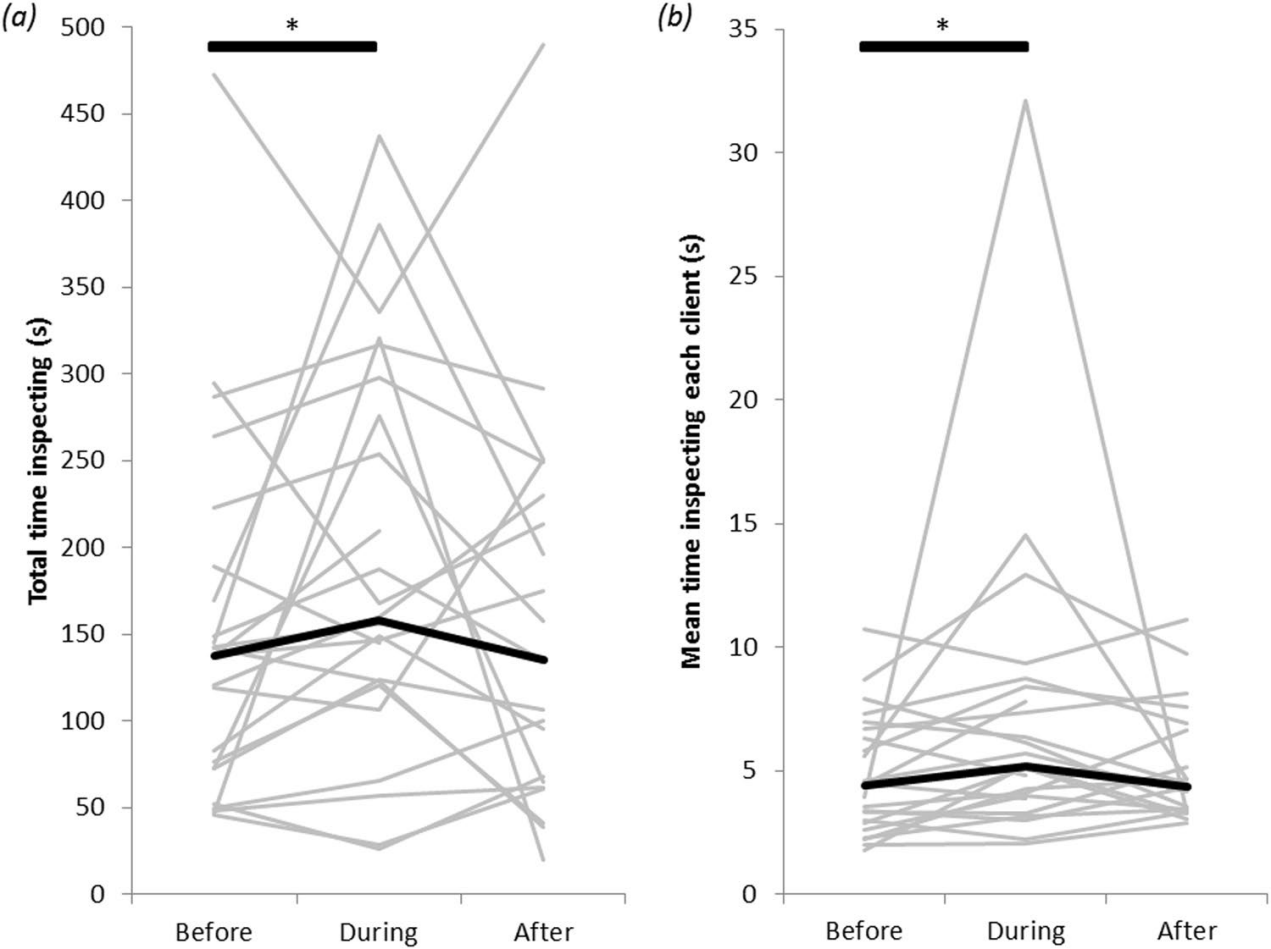
(**a**) Total time (in seconds) spent inspecting fish clients, and (**b**) average inspection duration (in seconds) per client, by juvenile bluestreak cleaner wrasses during 20-min observation periods before, during and after exposure to motorboat noise. Grey lines join the values from the same cleanerfish; black lines show the median values. N = 24 cleanerfish. Bars with stars indicate statistically significant differences.

Cleanerfish spent significantly longer (31 s of total inspection time, on average) inspecting clients during than before exposure to motorboat noise (Wilcoxon signed-rank test, V = 77.5, n = 24, p = 0.04; Fig. 2a), with 71% (17 of 24) of individual cleaners increasing their total inspection time. Total inspection duration declined after motorboat exposure, resulting in a level not significantly different from that observed either before (V = 88, n = 21, p = 0.35) or during (V = 155, n = 21, p = 0.18) motorboat exposure (Fig. 2a).

The longer total inspection duration was not due to heavier client traffic at cleaning stations (see above). Instead, cleaners spent ~1 s longer, on average, with individual clients during motorboat exposure than before (Wilcoxon signed-rank test, V = 68, n = 24, p = 0.018; Fig. 2b). Two thirds of cleaners (16 of 24) increased their time spent with each client. The decline in time inspecting each client after motorboat exposure was not significant (V = 159, n = 21, p = 0.14), but it reached a level that was not significantly different to that observed before motorboat exposure (V = 69, n = 21, p = 0.11; Fig. 2b).

#### Cooperation in cleaning interactions

**Figure 3:**
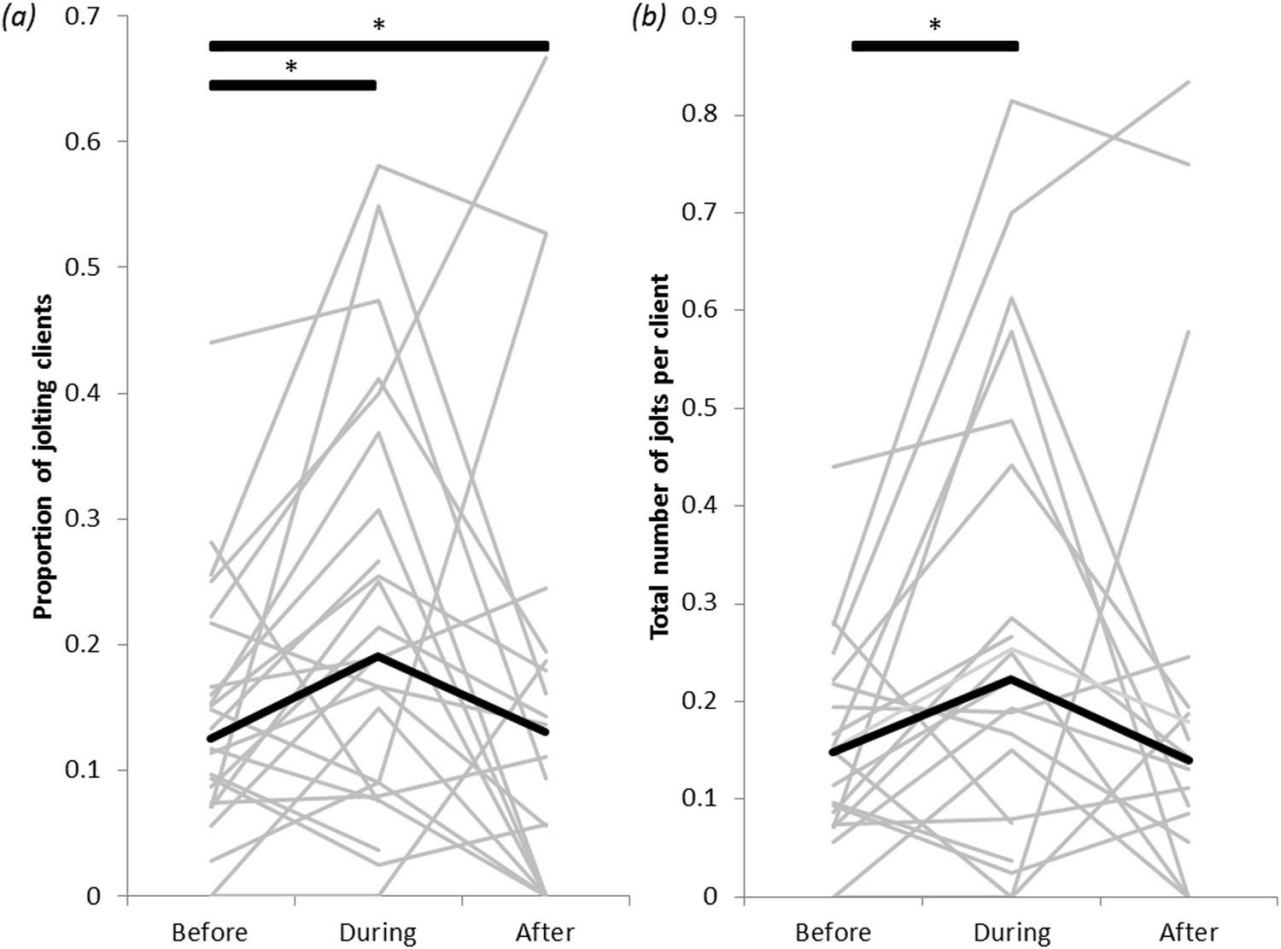
(**a**) Proportion of fish clients that jolted during inspections by juvenile cleaner wrasses and (**b**) number of jolts per client during 20-min observation periods before, during and after exposure to motorboat noise. Grey lines join results from the same cleanerfish; black lines show the median values. N = 24 cleanerfish. Bars with stars indicate statistically significant differences.

The proportion of clients that jolted in response to cleanerfish contact increased significantly, by 9% on average, during exposure to motorboat noise compared to the previous 20 min (Wilcoxon signed-rank test, V = 54, n = 24, p = 0.01; Fig. 3a); an increase was observed in 71% (17 of 24) of cleaners. This effect was short-lived: the proportion of jolting clients returned to initial levels after motorboat passes ceased (during vs. after: V = 171, n = 21, p = 0.05; before vs. after: V = 90, n = 21, p = 0.59; Fig. 3a). The number of jolts observed per client also increased during motorboat exposure, by nearly 50% on average (before vs. during: V = 142, n = 24, p = 0.02; Fig. 3b), but it remained elevated after motorboat noise ceased (during vs. after: V = 49, n = 21, p = 0.20; before vs. after: V = 126.5, n = 21, p = 0.08; Fig. 3b). Surprisingly, the higher proportion of jolting clients and higher rates of jolting during motorboat exposure did not result in a concomitant increase in proportion of clients that chased cleaners (before vs. during: V = 77, n = 24, p = 0.32; during vs. after: V = 77.5, n = 21, p = 0.64).

## Discussion

The effects of anthropogenic noise on individual animals are increasingly recognised^2, 3, 36^. We add altered mutualisms to the wide-ranging consequences of acoustic disturbances. When exposed to a passing motorboat, interactions between juvenile cleaner wrasses and their clients lasted longer, but more importantly, they were also less cooperative than prior to, or immediately after, the disturbance. Our results suggest that the integrity of an ecologically important mutualism on coral reefs^24, 25^ might be at risk in areas of heavy motorboat traffic.

Divers are noisy underwater. The regular breathing of a diver was easily detected on particle acceleration and pressure spectrograms recorded 1–2 m away, to simulate the distance of observers to cleaning stations (Figures S1 and S2). Diver noise and presence can cause some fish species to flee or hide, and higher fish abundances are sometimes recorded by divers using silent rebreathers instead of conventional SCUBA equipment (e.g. ref. 37). In our study, diver presence or noise might have affected fish behaviour, but there was no sign of shifts in cleanerfish or client behaviour over 60 min. Any effect of divers can therefore be considered to have been constant across the duration of our experiment. The behavioural changes seen in cleanerfish exposed to motorboat noise can therefore be clearly ascribed to a motorboat rather than to a diver effect. Indeed, the noise generated by a motorboat passing 10–30 m away was far louder across all frequencies than a diver much closer to the hydrophone. Whether it is the presence or noise of a motorboat that alters fish behaviour is difficult to tell (e.g. ref. 38), but the divers could neither see the motorboat nor feel its wake during the experiment. We do note the caveat that we used only two motorboats in our study and therefore speculate that our findings may be extrapolated to other engine types and motorboat conditions.

Acoustic disturbances increased inspection duration. In the presence of a motorboat, cleaners spent approximately 30 sec longer inspecting clients, i.e. a 25% increase in total inspection time compared to before and after boat exposure. This increase was not caused by an increase in the number of visiting clients inspected, or by the presence of a different suite of client species, e.g. large-bodied clients which usually have more ectoparasites and are inspected for longer^39, 40^. Instead, cleaners took slightly longer (1 sec) to inspect each client. This small difference scales up to approximately 18 additional minutes of inspection time per day (given ~90 clients per hr and 12 hr of cleaning activity daily), without an obvious foraging benefit, since the clientele composition remains constant. Longer inspections by cleaners are consistent with the idea of noise acting as a distraction^41^. Foraging animals exposed to noise might focus narrowly on small search areas^42^ or be distracted by non-food stimuli^43^, both of which can increase foraging time to maintain overall food intake. For example, in three-spined sticklebacks, brief exposures to white noise resulted in food-handling errors and poorer ability to discriminate between food and non-food items – behaviours that point to cognitive impairment due to divided and/or shifted attention^30, 44^. It is also possible that resource availability to cleaners in terms of ectoparasite visibility or ease of access on fish clients increased during motorboat exposure if noise affected parasite behaviour and/or distribution.

The second major change we observed in cleanerfish behaviour as a result of motorboat noise was decreased cooperation with clients. This was reflected by both a higher proportion of jolting clients and a higher number of jolts per client, with jolts indicating dishonest biting by cleaners^21, 45^. This shift could be caused by direct endocrine effects or be another consequence of cognitive impairment. Motorboat noise might act as a stressor to fishes, activating the hypothalamic–pituitary–interrenal axis to increase cortisol levels^46^, which can lead to increased intraspecific aggression in various social situations^27, 47^. In cleaner wrasses, experimental increases in cortisol levels are associated with a number of behavioural changes during cleaning interactions, including higher levels of dishonesty as indicated by higher jolting rates of clients^48^. However, if effects are due to cortisol increase, we would not anticipate a return to pre-exposure levels in the 20 min post-exposure, which we observed for the proportion of clients that jolted. Alternatively, noise can interfere with cognitive ability by overloading an animal’s attention capacity^41^. Foraging decisions by cleaner wrasses are highly sophisticated^32^ and whether to cheat or clean honestly depends on recognition of individual clients^31^ and recollection of the outcomes of previous interactions with specific individuals^19^. These abilities may be among the increasingly wide range of cognitively influenced activities now known to be impaired by noise (e.g. refs 30 and 41). Finally, it is also possible that cleaners took advantage of client distraction caused by motorboats (see below) and reduced their service quality. For example, cleaner wrasses immediately take mucus instead of ectoparasites if confronted with an anaesthetized client that cannot retaliate in response to cheating^49^.

Several client behaviours that are central to cleaning interactions were not affected by noise exposure. For example, client visits rates did not change with the onset of motorboat noise, nor did the proportion of clients initiating interactions by posing. However, the fact that increased cheating by cleaners did not result in clients leaving interactions sooner – cleaning interactions were in fact longer – or in more retaliatory chasing in response to cheating by cleaners, which they can do to enforce honesty^16^, supports the idea that clients were distracted.

Our results suggest that cleaning interactions are affected by motorboat noise. Short-term exposure to loud motorboats led to longer, but less cooperative inspections by cleaner wrasses. Two key questions have to be answered to gauge the importance of these findings for conservation and management. First, do these altered cleanerfish behaviours ultimately result in less effective ectoparasite removal? Second, can cleaners and their clients habituate to motorboat noise? There is evidence of increased tolerance to chronic noise over time in coral reef fishes^50^ and other fish species^51^, which could mitigate the broader ecological impacts of less effective cleaning interactions. However, it is not clear whether all parties in cleaning interactions have the same capacity to habituate to noise. In the absence of habituation, only local regulation – e.g. the creation of marine protected areas with enforced no-go restrictions – can reduce the impact of vessel noise on these important mutualistic interactions.

## Materials and Methods

### Study sites

This study was approved by Simon Fraser University, Canada. All procedures performed in accordance with the legal requirements for animal research in Canada, and were conducted under a permit (#1124B-14) issued by Simon Fraser University Animal Care Committee. The study was conducted at nine lagoonal coral reef sites on the north shore of Moorea, French Polynesia. The sites varied in maximum depth (1.5–8 m) and topography, and were chosen because all allowed for safe, repeated passes of a motorboat near the observation sites.

### Behavioural observations

#### Habituation to diver presence and noise

We first conducted a pilot study over 2 days to examine potential habituation by fish engaged in cleaner–client interactions to the presence and noise of a SCUBA diving observer. Four observers watched a total of 12 juvenile cleaner wrasses at one site, which was not part of the main study. We studied juveniles for two reasons: (1) at this location, they are more active cleaners than adults (IMC, SCM, personal observations) and (2) their cleaning behaviour is not yet influenced by sexual motivation, making it more consistent across individuals. Each cleaner was observed, in the absence of any motorboat noise, by a motionless diver hovering or kneeling on the sand 2–3 m (horizontal distance) away for three successive 20-min periods, which mirrors the experimental treatment (see below). During each 20-min period, we recorded the species identity of each visiting client and duration of each inspection (i.e., when the cleaner surveys the body surface, gills or buccal cavity of the fish client).

#### Effects of motorboat noise on cleaner–client interactions

Following the pilot study, we searched for juvenile cleaner wrasses at eight additional sites, and each diver observed the first cleaner he/she encountered. All observations lasted 60 min, divided into three 20-min periods: (1) a pre-exposure, control period (no motorboat noise); (2) an exposure period (motorboat noise present); and (3) a post-exposure period (no motorboat noise). At the end of the pre-exposure period, all diving observers released a tethered surface marker buoy to signal the start of the exposure period. A research assistant drove (estimated speed: 4–10 knots) one of two small outboard motorboats (boat with 25 hp engine chosen randomly on each day) multiple times past the marker buoys, no closer than 10 m from the buoy (for diver safety) and no further than 100 m. This treatment was expected to generate noise at a broad range of frequencies including 1–3000 kHz, which is audible to most fish species^52^, at levels of 80–150 dB re 1 μPa (RMS, full spectra), which have been shown to affect behaviour in reef fish (e.g. ref. 6). We recorded the experimental motorboat noise underwater to measure the actual range of frequencies and levels generated around a cleaner (see below).

During each of the three 20-min periods, we recorded the number and species of fish visiting the focal cleaner and the duration of each inspection. We also noted whether the interaction was initiated by the client by adopting a stereotyped incitation posture, which increases the likelihood of being inspected^35^, whether clients jolted during the interaction, and whether clients chased the cleaner. We moved to a new site after each observation to ensure that cleanerfish were only observed once. Because fish were not marked, a few individual clients might have contributed to the clientele of more than one cleanerfish; however, this is unlikely to have occurred often given the high density of fish at the study sites. Other boats were not observed or heard during observations.

### Motorboat noise recording and analysis

#### Acoustic recordings

To evaluate and compare the range of frequencies and sound levels generated by the diving observer and the passing motorboat during our experiment, we deployed recording equipment ~50 cm from one juvenile cleaner wrasse (which was not observed in the main study) at 4 m depth and a diver with a surface marker, posing as an observer, ~2 m away (horizontal distance). One of two field assistants then drove one of the two motorboats used in our experiment repeatedly near the diver, in an accurate simulation of our experimental treatment. Since some fish can detect pressure and all fish detect particle motion, we recorded both sound pressure and particle acceleration before and during multiple motorboat passes conducted in a 5-min period. Sound pressure was recorded using an omnidirectional hydrophone (HiTech HTI-96-MIN with inbuilt preamplifier; sensitivity -165 dB re 1 V/μPa; frequency range 2 Hz–30 kHz; High Tech Inc., Gulfport MS), and particle acceleration using an accelerometer (M20L, sensitivity 0–3 kHz, manufactured and calibrated by GeoSpectrum Technologies, Dartmouth, Canada). Recordings were made on a laptop via a USB soundcard (MAYA44, ESI Audiotechnik GmbH, Leonberg, Germany). The USB sound card was fully calibrated using pure sine wave signals generated in SAS Lab (Avisoft, Germany), played on a MP3 player, measured in line with an oscilloscope.

#### Acoustic analysis

Acoustic recordings were analysed in MATLAB v2010a. Fast-Fourier transforms were used to transform time domain recordings into the frequency domain before power spectral density was calculated, to allow comparison of sound levels for each treatment across the frequency range 0–3 kHz. Sound levels are only considered up to 3 kHz due to the upper limit of the sensitivity of the accelerometer. This frequency range is likely to cover the hearing range of most fishes^52^.

### Statistical analysis

For our initial study of habituation to diver presence, we compared one client-focused measure, i.e. the number of client visits, and one cleaner-focused measure, i.e. total inspection duration (obtained by summing the durations of all inspection events for each cleaner), across the three consecutive 20-min observation periods. These measures did not meet the assumptions of parametric testing, even after transformation; therefore, we used non-parametric Wilcoxon’s signed-rank tests with continuity correction for pairwise comparisons of 20-min periods to account for the repeated-measures nature of our observations.

To examine the effect of motorboat noise, we compared seven behavioural metrics of cleaner–client interactions between the 20-min pre-exposure, motorboat-noise exposure and post-exposure periods. The measures were: (1) the number of client visits; (2) total inspection duration (calculated as described above); (3) the proportion of interactions initiated by clients; (4) inspection duration per client; (5) the proportion of jolting clients; (6) the number of jolts per client; and (7) the proportion of jolting clients that chased the cleaner in retaliation. None of these measures met the assumptions of parametric testing, even after transformation, so we used non-parametric Wilcoxon’s signed-rank tests with continuity correction for pairwise comparisons of 20-min periods to account for the repeated-measures nature of our observations. On three occasions where cleaners could not be found by divers after the motorboat exposure, we could only compare the pre- and during exposure periods.

To examine differences in cleaner clientele over time in both the habituation study and the motorboat-noise experiment, we used a permutation-based, non-parametric multivariate analysis of similarity (ANOSIM) using the software PRIMER (Plymouth Routines in Multivariate Ecological Research v. 6.1.13; PRIMER-E Ltd, Plymouth Marine Laboratory, Plymouth, UK)^53^. We created a visit frequency matrix (client species by cleaner/time period), square-root-transformed the data to reduce the influence of very abundant client species, and computed Bray-Curtis similarity coefficients between pairs of cleaners/time periods^54^. The ANOSIM procedure was carried out on the similarity matrix. ANOSIM generates an R statistic, which varies between 0 (similarities within and between samples are the same) and 1 (all samples within groups are more similar to each other than to any sample across groups) and is tested for difference from zero with a permutation test (in this study, N = 999 permutations). In the habituation study, we used a one-way ANOSIM to compare clientele among the three consecutive 20-min observation periods; in the motorboat-noise experiment, we used a two-way ANOSIM with motorboat exposure (three groups: before, during and after) and site (eight levels) as main factors. We visualized the differences in client assemblages among time periods with non-metric multidimensional scaling (MDS) plots, in which samples that are more similar in community composition appear closer together than more dissimilar samples. The extent of spatial distortion needed to represent community composition differences in two dimensions is estimated by MDS stress values. Stress values of <0.1 suggest that distances among samples in an MDS plot accurately reflect the extent of community differences^54^.

### Ethics

This study was approved by Simon Fraser University, Canada. All procedures performed in accordance with the legal requirements for animal research in Canada, and were conducted under a permit (#1124B-14) issued by Simon Fraser University Animal Care Committee.

### Data accessibility

Data are available as supplementary material.

## Electronic supplementary material

Supplementary Information 

Supplementary Dataset 1

Supplementary Dataset 2

Supplementary Dataset 3

Supplementary Dataset 4
