# Supplementary material for: Motorboat noise disrupts co-operative interspecific interactions
Source: Sci Rep. 2017 Aug 1;7:6987. doi: 10.1038/s41598-017-06515-2 (PMC5539144; doi:10.1038/s41598-017-06515-2)
Supplement: Supplementary file 1 — Supplementary Information [file 41598_2017_6515_MOESM1_ESM.pdf]

Supplementary Materials

Motorboat noise disrupts co-operative interspecific interactions

Sophie L. Nedelec, Suzanne C. Mills, Andrew N. Radford, Ricardo Beldade, Stephen D. Simpson, Brendan Nedelec and Isabelle M. Côté

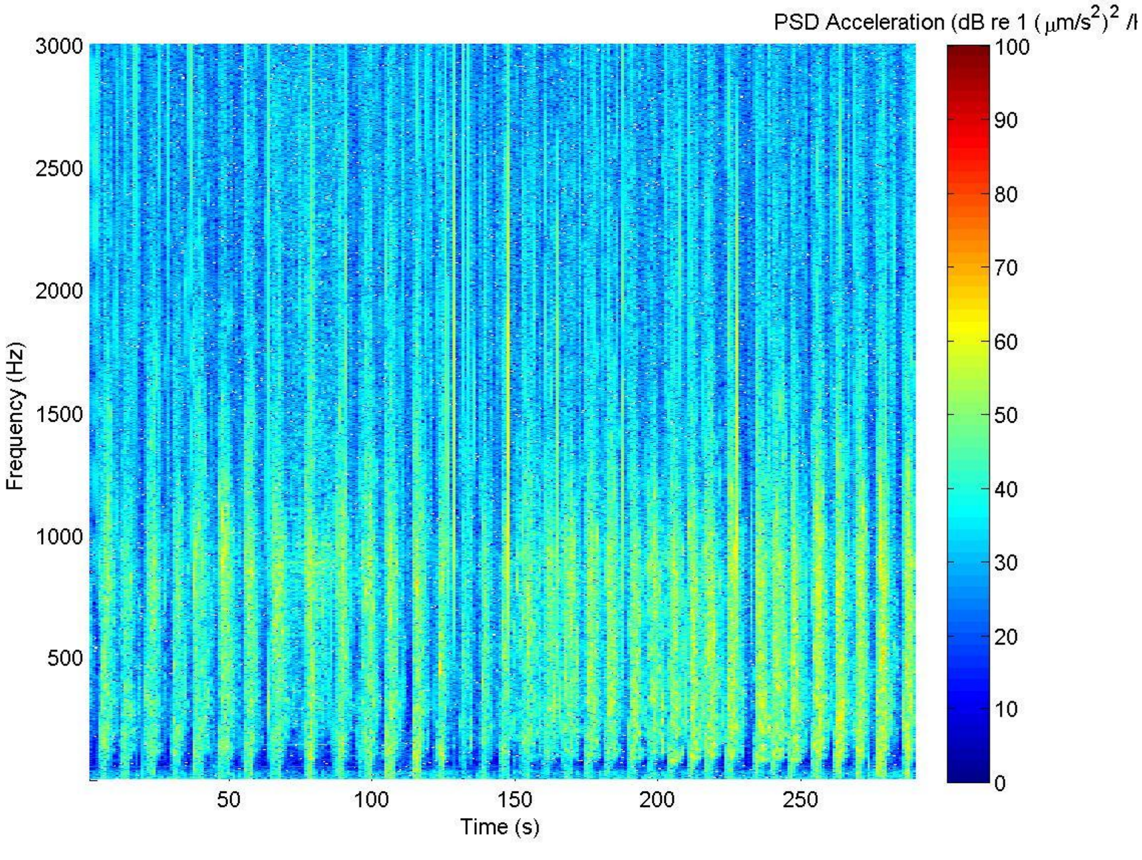

Figure S1. Monoaxial (x-axis – horizontal) particle acceleration spectrogram of a recording made at 4 m depth, 50 cm from a bluestreak cleaner wrasse on a coral wall with a SCUBA diver 1-2 m away. FFT length = 44100, time window = 1 s.

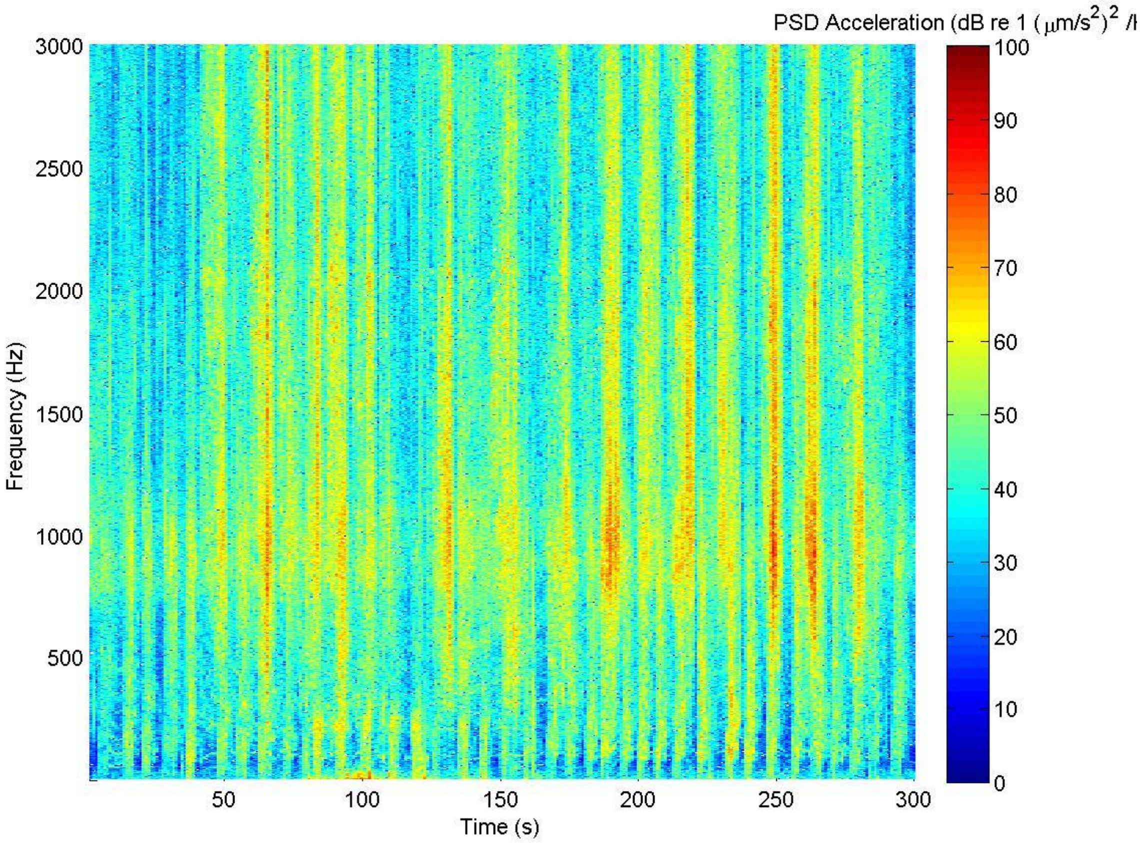

Figure S2. Monoaxial (x-axis – horizontal) particle acceleration spectrogram recorded 50 cm from a juvenile bluestreak cleaner wrasse at 4 m depth on a coral wall with a SCUBA diver present 1–2 m away while an outboard motor boat with a 40 hp engine was driven past multiple times at 10–50 m. FFT length = 44100, time window = 1 s.

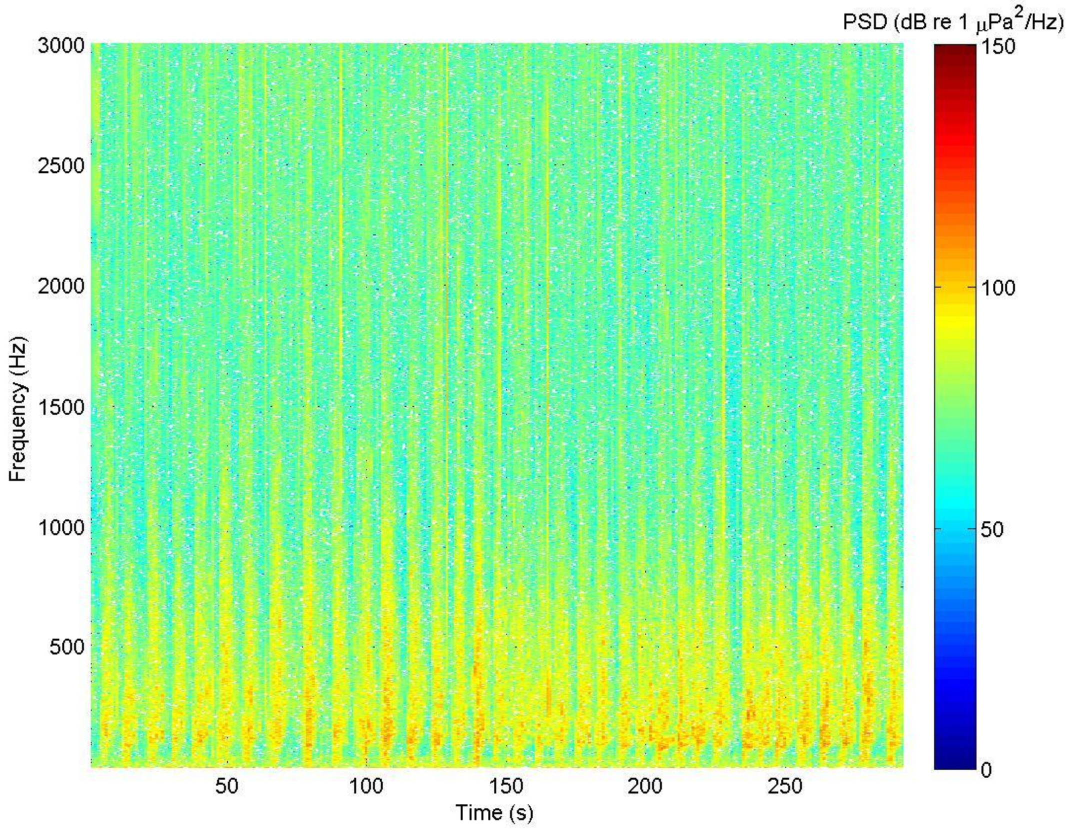

Figure S3. Spectrogram of sound pressure recording made at 4 m depth, 50 cm from a bluestreak cleaner wrasse on a coral wall with a SCUBA diver 1–2 m away. FFT length = 44100, time window = 1 s.

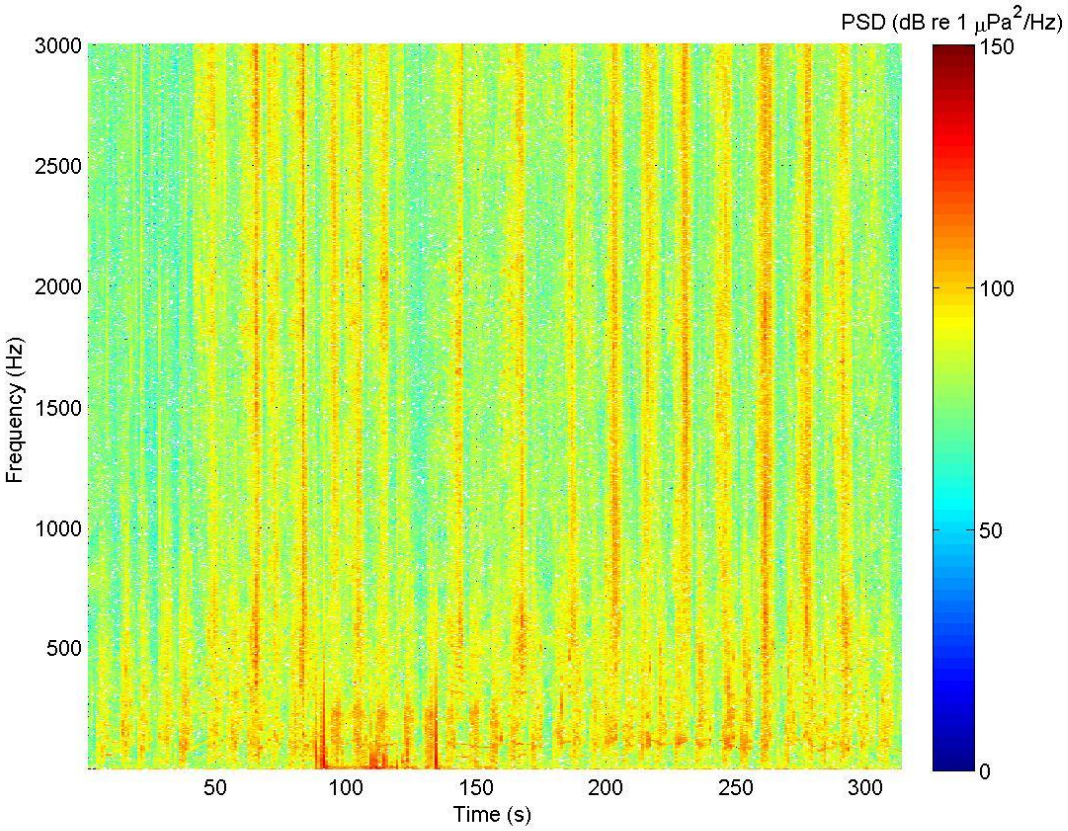

Figure S4. Spectrogram of sound pressure recorded 50 cm from a juvenile bluestreak cleaner wrasse at 4 m depth on a coral wall with a SCUBA diver present 1–2 m away while an outboard motor boat with a 40 hp engine was driven past multiple times at 10–50 m. FFT length = 44100, time window = 1 s.

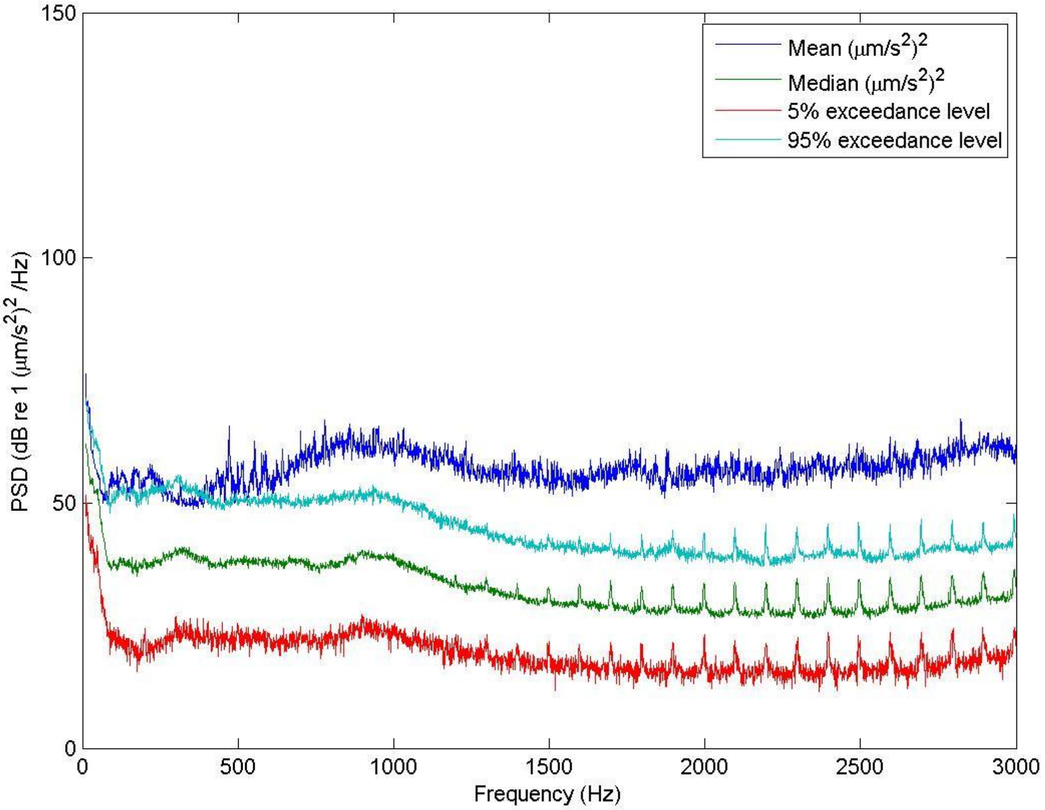

Figure S5. Power spectral density of monoaxial (x-axis – horizontal) particle acceleration recorded 50 cm from a juvenile bluestreak cleaner wrasse at 4m depth on a coral wall with a SCUBA diver present 1–2 m away. Mean, median and exceedance levels are calculated from 290 x 1 s samples using a FFT length equal to the sampling rate of the recording (44100).

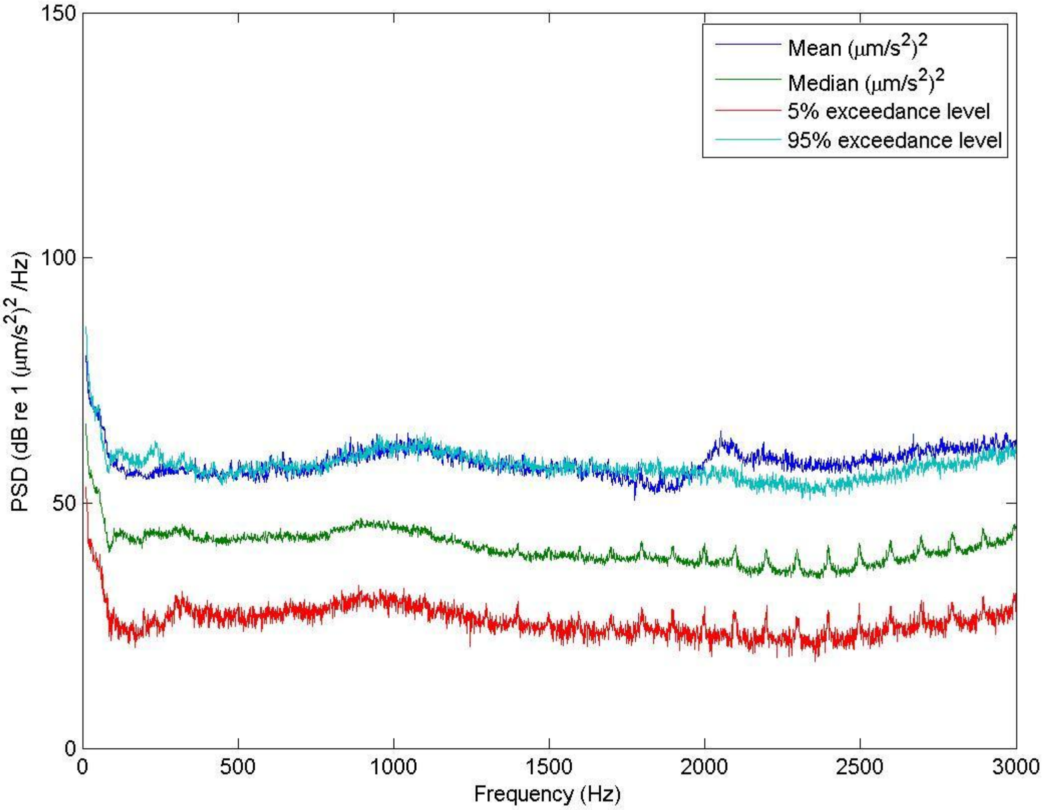

Figure S6. Power spectral density of monoaxial (x-axis – horizontal) particle acceleration recorded 50 cm from a juvenile bluestreak cleaner wrasse at 4m depth on a coral wall with a SCUBA diver present 1–2 m away while an outboard motor boat with a 40 hp engine was driven past multiple times at 10–50 m. Mean, median and exceedance levels are calculated from 300 x 1 s samples using a FFT length equal to the sampling rate of the recording (44100).

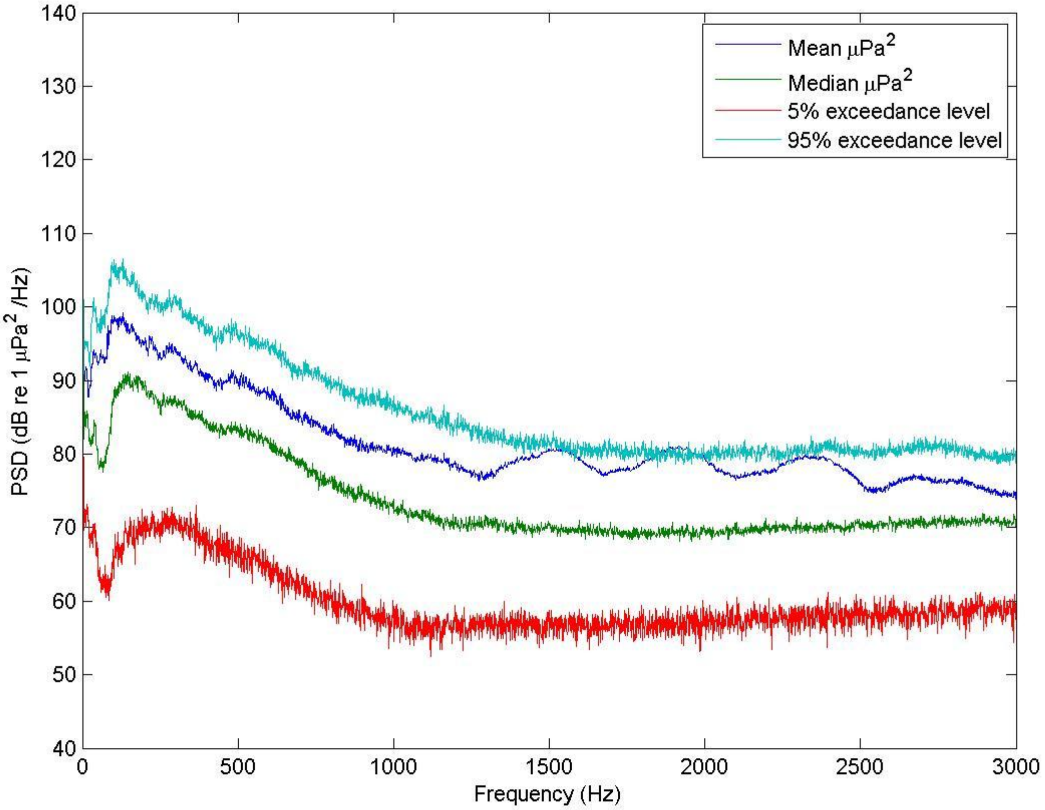

Figure S7. Power spectral density of sound pressure recorded 50 cm from a juvenile bluestreak cleaner wrasse at 4m depth on a coral wall with a SCUBA diver present 1–2 m away. Mean, median and exceedance levels are calculated from 290 x 1 s samples using a FFT length equal to the sampling rate of the recording (44100).

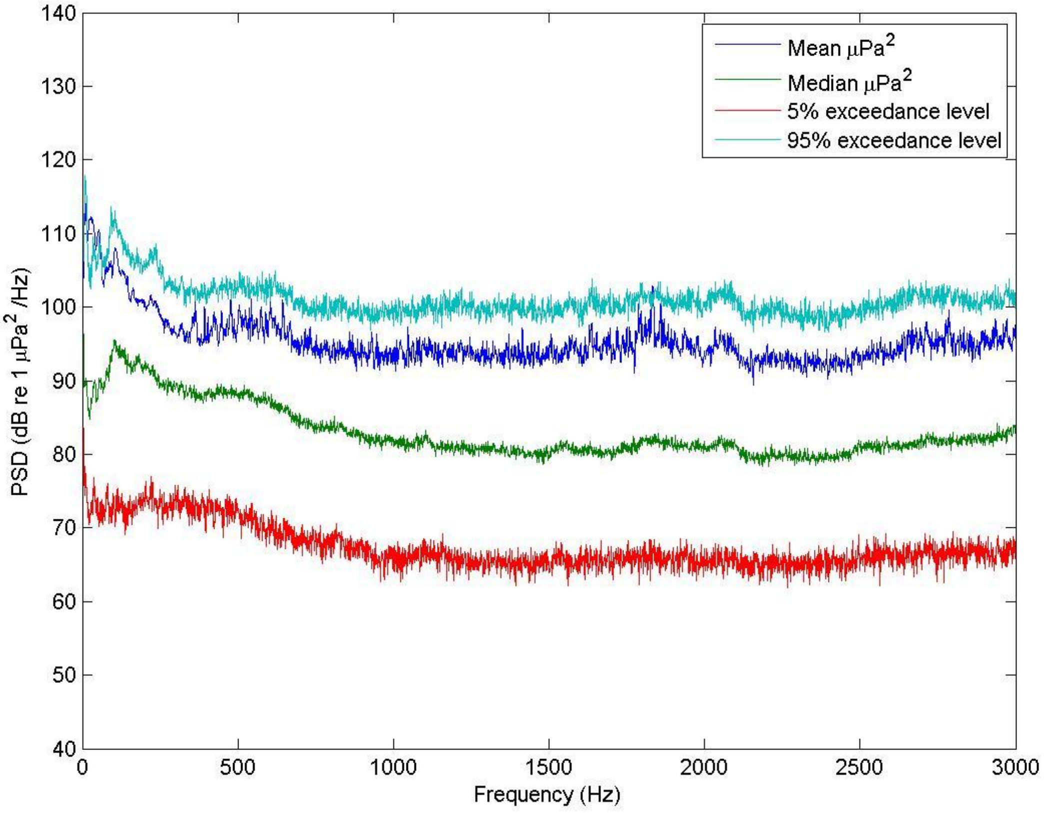

Figure S8. Power spectral density of sound pressure recorded 50 cm from a juvenile bluestreak cleaner wrasse at 4m depth on a coral wall with a SCUBA diver present 1–2 m away while an outboard motor boat with a 40 hp engine was driven past multiple times at 10–50 m. Mean, median and exceedance levels are calculated from 300 x 1 s samples using a FFT length equal to the sampling rate of the recording (44100).
